# Adaptive genetic algorithm based deep feature selector for cancer detection in lung histopathological images

**DOI:** 10.1038/s41598-025-86362-8

**Published:** 2025-02-08

**Authors:** Avigyan Roy, Priyam Saha, Nandita Gautam, Friedhelm Schwenker, Ram Sarkar

**Affiliations:** 1 Department of Computer Science and Engineering, Jadavpur University, Kolkata, India; 2 Institute of Neural Information Processing, Ulm University, Ulm, Germany

**Keywords:** Computational science, Computer science, Information technology, Scientific data, Software, Statistics

## Abstract

Cancer is a global health concern because of a significant mortality rate and a wide range of affected organs. Early detection and accurate classification of cancer types are crucial for effective treatment. Imaging tests on different image modalities such as Histopathology images, provide valuable insights into the cellular and architectural features of tissues, allowing pathologists to make diagnosis, determine disease stages, and guide treatment decisions. They are an essential tool in the study and understanding of diseases, aiding in research, education, and patient care. Convolutional neural network based pretrained deep learning models can be used successfully to detect lung cancer. In this study, we have used a channel attention-enabled deep learning model as a feature extractor followed by an adaptive Genetic Algorithm (GA) based feature selector. Here, we calculate the fitness score of each chromosome (i.e., a candidate solution) using a filter method, instead of a classifier. Further, the GA optimized feature vector is fed to the K-nearest neighbors classifier for final classification. The proposed method shows a promising result with an overall accuracy of 99.75% on the LC25000 dataset, which is a publicly available dataset of lung histopathological images. The source code for this work can be found https://github.com/priyam-03/GA-Feature-Selector-Lung-Cancer.

## Introduction

Cancer^1^ is the second leading cause of death worldwide. The National Cancer Institute (NCI) defines cancer as a condition in which aberrant cells multiply uncontrollably and have the potential to infect neighboring tissues. Any human body organ could be affected by cancer, however, the colon, lungs, liver, breasts, rectum, brain, prostate, stomach, and skin are the most frequently affected organs. Lung cancer is one of the most frequent malignancies that kills both men and women equally. Pain, fatigue, nausea, persistent cough, shortness of breath, weight loss, muscle aches, bleeding, and bruises are a few of the signs of cancer. However, neither are they universal among patients nor are any of these symptoms particular to cancer. As a result, cancer detection becomes challenging without a thorough biopsy, Positron Emission Tomography (PET)^2^ scan, Computed Tomography (CT)^3,4^ scan, Magnetic Resonance Imaging (MRI) scan, or ultrasound imaging. According to research, some people are born with the cancer-causing gene. Such individuals must regularly undergo diagnostic procedures, which are mostly expensive. The World Health Organization (WHO) reports that over 70% of cancer-related fatalities occur in low and middle-income countries. In such a situation, setting up healthcare facilities and engaging with the public becomes quite a challenge. This issue can be resolved by applying artificial intelligence (AI) and deep learning based models to identify the type and sub-type of cancer as well as whether or not it has started. In this work we have employed the LC25000^5^ dataset consisting of 15000 histopathological images of lung tissue. We have selected histopathological images because histopathological images provide more comprehensive information than CT scan^3^ images. Deep learning has been used in place of machine learning because deep learning models can automatically learn better feature representations of the input data. They can identify complex patterns of the raw data without relying on explicit feature engineering^6^. This eliminates the need for handcrafted features and allows the model to extract relevant features directly from the data. They can capture intricate patterns, non-linear relationships, and subtle dependencies in the data, leading to improved performance in tasks like image classification, speech recognition, and natural language understanding to name a few. However, the feature maps generated by any deep learning model may have some redundancy, which can be dealt with by using a feature selection (FS) approach^7^^8^.

Considering the aforementioned points, in this paper, we have proposed a model, which classifies the sample images as benign, adenocarcinoma, or squamous cell carcinoma using histopathological images of lung cancer tissue. Initially, we apply a channel attention^9^ enabled DenseNet121^10^ CNN model^11^ as a feature extractor. After that the dimension of the extracted features is reduced by a filter methods aided GA^12^ based feature selection mechanism. The final classification is performed using a KNN^13^ classifier. The reason why KNN classifier has been used is because it is not only lightweight, which is a primary aim for this work, but also provides promising results if we compare with other classifiers. This fact has been demonstrated in an article by Naskar et al.^14^

Our key contributions can be summarized as follows:

- We propose a channel attention enabled DenseNet121 CNN model as the feature extractor.
- The extracted features are fed into an adaptive GA based feature selector. The final classification is done by a KNN classifier.
- Our proposed method is trained and tested on the 15000 lung images of the LC25000 dataset. The model achieves a satisfactory level of accuracy of 99.75% which is higher than most contemporary methods proposed earlier.
- Our work employs GA in an adaptive way, which leads to consumption of lesser resources and also makes execution faster than traditional wrapper based GAs, where classifiers are used to calculate the fitness value of chromosomes.

The rest of the paper is organized as follows: Related work found in the literature is discussed in Section “Related work”. In Section “Dataset description”, we discuss the dataset used in this research. The proposed model is described in Section “Proposed method”. The experiments and results are summarized in Section “Experiments and results”. Section “Discussion” consists of the discussions on the strengths and limitations of the model. Finally, the paper is concluded in Section “Conclusion and future scope” along with the future scopes of this work.

## Related work

We have divided this section into two parts- (a) Work on Lung Cancer (b) Work involving the use of GA.

### Work on lung cancer

In this section, we discuss some recently published work in the domain of lung cancer detection. It has been observed that mostly researchers have applied deep learning based methods to solve this problem. Gopi Kasinathan et al.^15^ proposed a Cloud-based Lung Tumor Detector and Stage Classifier (Cloud-LTDSC) as a hybrid technique. The employed dataset is the LIDC-IDRI dataset^16^ which consists of CT scan / PET scan images. The model achieved 97% accuracy. Amitava Halder et al.^17^ put forward a 2-Pathway Morphology-based Convolutional Neural Network (2PMorphCNN) and used the LIDC-IDRI dataset^16^. The proposed model achieved an accuracy of 96.10%.

Shahid Mehmood et al.^18^ proposed an image contrast enhancement based approach involving class selective image processing (CSIP) which uses a pre-trained AlexNet model for classification to predict lung cancer. The LC25000 dataset was used. The proposed model achieved 89.9%, precision of colon-aca, colon-n, lung-aca, lung-n, lung-scc, are 98.8%, 99.7%, 67.4%, 99.9%, and 96% respectively. Recall values of colon-aca, colon-n, lung-aca, lung-n, and lung-scc are 99.1%, 99.9%,97.1%,99.8%, and 53.3% respectively. Masud et al.^19^ employed the LC25000^5^ dataset and applied image enhancement techniques to reduce noise, improve particular attributes, and extract important information from images, making them better suited for the learning process. 2D Fourier and 2D Wavelet features were extracted as part of the feature extraction process, which was followed by the development of feature sets. The generated feature set was then input into a single channel CNN that had a batch normalization layer with a dropout rate of 0.3, two max-pooling layers, and three interleaved 2D convolution layers. The maximum testing accuracy was 96.33%, and the highest training accuracy was 98.91%.

Mangal et al.^20^ used the LC25000 dataset and a single channel CNN composed of a convolution layer, a pooling layer, and a flatten layer to turn the output from the preceding layer into a 1D tensor. A fully connected layer was then inserted into the flattened tensor. On data related to lung cancer, the model produced training accuracy of 97.92%, validation accuracy of 97.90%, and accuracy of 96.95% and 96.61%, respectively, on data related to colon cancer. The CNN model, utilized by Hatuwal et al.^21^ to classify images, was built using a liner stack of convolutional layers with fully linked layers, max pooling, and kernel filters were applied to training and test images. The given object was classified using the softmax function. A neural network was used that included three hidden layers, one input layer, and one fully connected layer. Each convolutional layer used a kernel matrix of (3, 3) with ReLU(x) = max (0, x) as the activation function. In order to decrease the computation parameters in the next convolution layer, a max pooling size of (2, 2) was implemented. For the model, a dropout value of 0.1 was used. The model achieved a training accuracy of 96.11% and a validation accuracy of 97.20% in the final epoch.

Nishio et al.^22^ proposed a homology-based image processing approach for lung cancer detection. Their method involved texture analysis for feature extraction, specifically using betti numbers^23^. By training a machine learning model on the LC25000 dataset, including lung adenocarcinoma, benign, and squamous cell cancer images, they achieved a notable test accuracy of 99.43%.

### Work involving GA

GA is one of most popular optimization algorithms used in various research fields over the years. Liu et al.^24^ used GA to train neural network for land cover classification. Their approach included a real coded GA strategy, hybrid with Back Propagation (BP) algorithm. The model was able to achieve 97% accuracy on the SPOT-4 XS imagery of Jiangning County, Jiangsu, China. Singh et al.^25^ proposed a semi supervised method for satellite image classification based on GA and Radial Basis Function Neural Network (RBFNN). The Landsat 8 OLI dataset was used for this work. Initially the features were extracted from preprocessed images by spectral indices. The extracted features were fed into GA using which the RBFNN was trained till the termination conditions were met. The proposed method managed to achieve an accuracy of 94.92%.

Tsai et al.^26^ employed Gaussian-distributed fuzzy membership functions (GDMFs) for their study. The GDMFs were initially generated using various texture-based features obtained from reference images. Subsequently the shapes of GDMFs were optimized by a GA learning process. After optimization, the classifier is used for disease discrimination. The proposed model aachieved an average accuracy of 96% for myocardial heart disease and accuracy of 88.5% at 100% sensitivity level for microcalcification on mammograms. Zhang et al.^27^ introduced a novel approach that combines a neural-genetic algorithm with a neural network classifier for feature selection. Their research focused on the classification of various small breast abnormalities by incorporating computer-extracted statistical features from mammograms along with manually extracted features. The results demonstrated promising accuracy rates of 90.5% for calcification cases and 87.2% for mass cases using different feature subsets.

Ali et al.^28^ proposed a hybrid approach that combines filter-based feature selection methods and a genetic algorithm to improve cancer classification accuracy on high-dimensional microarray datasets. The method applies filter feature selection techniques, such as information gain, information gain ratio, and Chi-squared, to identify significant features. A genetic algorithm then optimizes and enhances the selected features, showing superior performance in Accuracy, Recall, Precision, and F-measure compared to conventional machine learning methods on breast, lung, central nervous system, and brain cancer datasets. Sarkar et al.^29^ introduced Diversification of Population in GA (DPGA), an FS algorithm that reduces the number of features extracted by a modified texture-based feature descriptor, achieving good outcomes on a 7-class microstructural image dataset. Lin et al.^30^ proposed a GA-based FS method for image retrieval and classification, reducing features and increasing accuracy at the expense of computational cost using adaptive motifs co-occurrence matrix (AMCOM), gradient histogram for adaptive motifs (GHAM), and adaptive color histogram for K-means (ACH).

Sarkar et al.^31^ proposed a model that encoded sensor data time series as multi-channel images using continuous wavelet transform. A spatial attention-aided CNN extracted higher-dimensional features, and a novel FS method combined with Mutual Information, Relief-F, and mRMR filters was used to identify important features for human activity recognition. Guha et al.^32^ introduced the cooperative genetic algorithm (CGA), incorporating game theory concepts to enhance classification accuracy and time efficiency in human activity recognition. CGA showed significant improvement in overall classification accuracy using a small fraction of the original feature vector.

GA has also been used successfully in various fields such as numerical recognition system for Devanagari , Bangla and Roman scripts by Chowdhury et al.^33^, proposal of a new method called Deluge based GA (DGA) by Guha et al.^34^, proposal of a novel model, named binary genetic swarm optimization (BGSO), which initially allows GA and Particle Swarm Optimization (PSO) to run independently by Ghosh et al.^35^, handwritten word recognition by Malakar et al.^8^, cancerous gene identification by Ghosh et al.^36^, gene selection from microarray data by Marjit et al.^37^, breast cancer detection in thermograms by Pramanik et al.^38^. Some more methods include stock prediction based on GA based FS by Chen et al.^39^, stock market prediction using GA-optimized multi-channel CNN by Chung et al.^40^, parameter optimization of solar PV cell/module using GA based on non-uniform mutation by Saadaoui et al.^41^, GA based optimized leach protocol for energy efficient wireless sensor networks by Bhola et al.^42^. The success of these methods as well as the varied applications of GA makes it a promising algorithm for FS.

## Dataset description

**Fig. 1:**
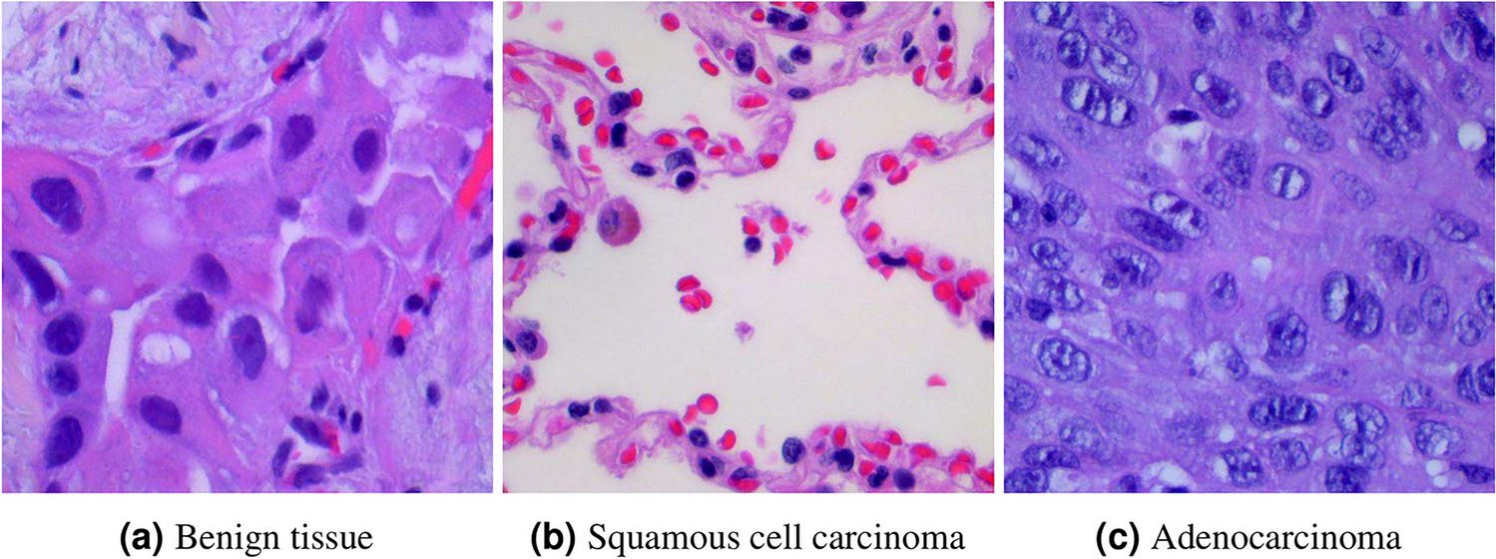
Samples from the three different classes of lung cancer histopathological images taken from the dataset LC25000.

The dataset used in this study, contains nearly 15,000 histopathological images with 3 classes of lung cancer consisting of benign tissue, squamous cell carcinoma and adenocarcinoma. It has been taken from the LC25000 dataset, which is available as a public data repository for lung and colon cancer images. This dataset comprises approximately 15,000 sample images of lung cancer and 10,000 sample images of colon cancer. All images are 768$\times$768 pixels in size and are in jpeg file format. For our work, we have only taken the lung adenocarcinoma, lung squamous cell carcinoma, and benign lung tissue images each numbering 5000 i.e., we have a total of 15,000 images. The details have been tabulated in Table 1. Three sample images, one from each class, have been shown in Fig. 1.

**Table 1:** Details of the lung histopathological dataset.

Split	ACA	N	SCC	Total
Train	3500	3500	3500	10,500
Test	1000	1000	1000	3000
Val	500	500	500	1500
Total	5000	5000	5000	15,000

## Proposed method

**Figure 2:**
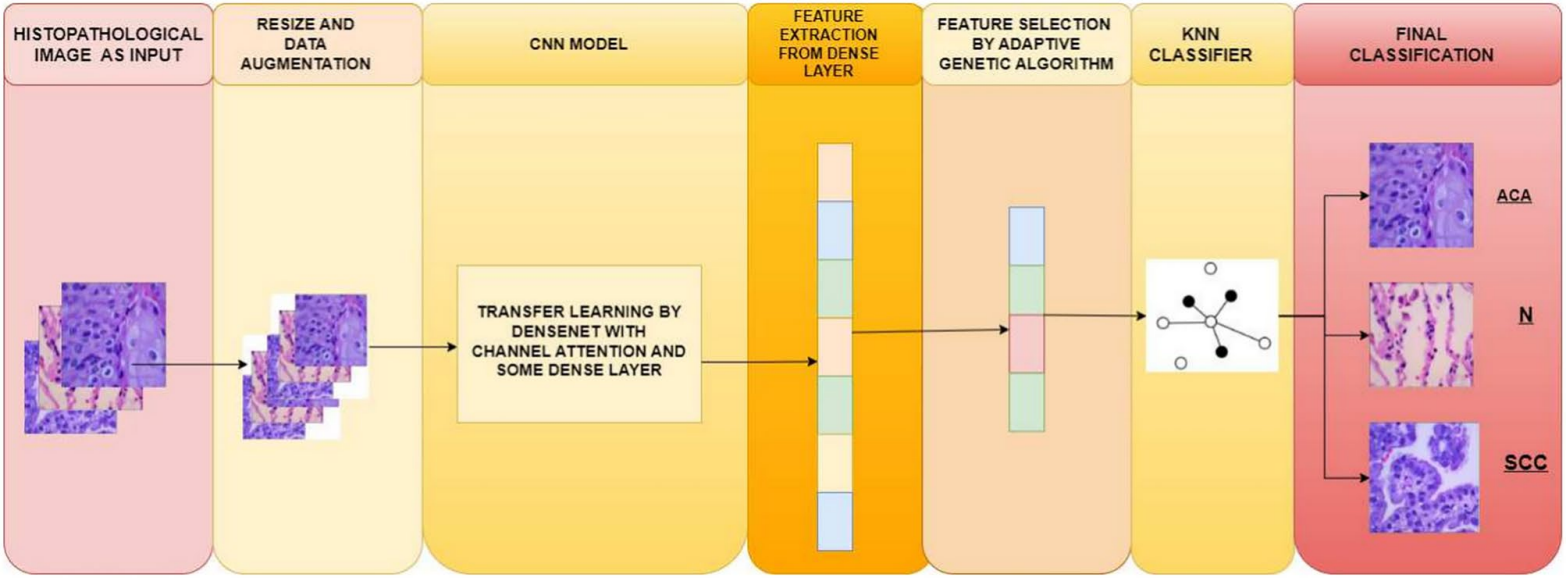
Overall workflow of proposed method developed for lung cancer classification using histopathological images.

In this work, we have proposed a lung cancer classification model combining the concepts of deep learning and GA. Figure 2 shows the overall workflow of the proposed method. Each of the steps has been discussed in details in the following sub-sections.

### Data preprocessing

In this study, we perform data preprocessing on a lung image dataset for classification tasks. The training dataset is divided into training and validation subsets. To enhance the model’s performance and generalize better, we apply several preprocessing techniques. In the training data, we first rescale the pixel values by dividing them by 255. This step ensures that all images have consistent pixel intensity values. We also use different data augmentation techniques, including random rotations of up to 20 degrees and horizontal flipping of the images. These augmentations aim to increase the diversity of the training data and help the model learn robust features.

The training set is then organized into batches of 16 images and resized to a target size of $224\times 224$ pixels. The images are represented in RGB color mode to capture the color information. The corresponding labels are encoded in a categorical format, enabling multi-class classification. For the validation data, we apply a similar preprocessing pipeline, including rescaling and resizing to the same target size. However, we do not apply data augmentation to the test set to ensure an unbiased evaluation of the model’s performance. By performing these preprocessing steps, we prepare the dataset in a standardized and augmented manner, enabling effective training and evaluation of the model for lung image classification tasks.

### Feature extraction using CNN

A CNN is a substantial deep neural network that models and recognizes stimuli as the brain’s visual cortex processes them. One might see a basic CNN model like a mix of the features extractor and the classifier components. The CNN’s feature extractor, which consists of a sequence of convolution layers followed by pooling layers, is one of the hidden layers. It aims to discover intricate characteristics and patterns specific to a image class after convolving using a variety of filters. Finally, the classification component makes use of these features.

#### DenseNet121

**Fig. 3:**
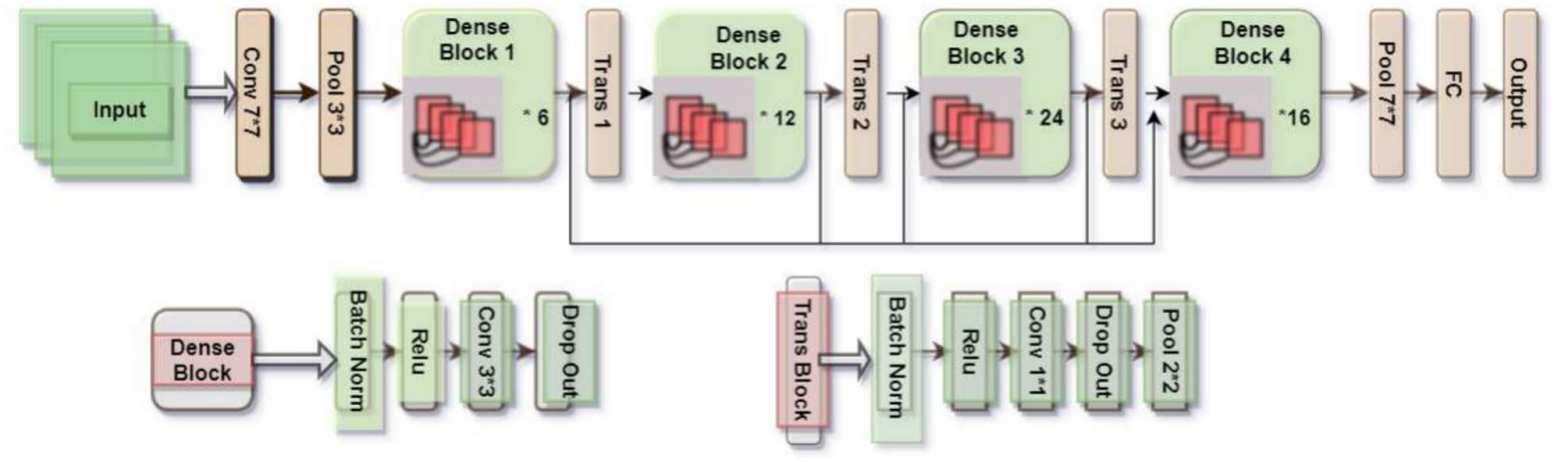
An illustration of the DenseNet121 model showing four dense blocks. The omitted blocks follow the same structural pattern as the illustrated ones, excluded for space considerations.

The DenseNet121 model has 121 layers and uses a dense block structure, where each layer is connected to all previous layers in a feedforward manner. DenseNet121 is composed of six dense blocks, each consisting of an equal number of sub-layers. These sub-layers include batch normalization, ReLU activation function, and convolution layers. The dense blocks are then followed by transition layers, which utilize $1\times 1$ convolutions and $2\times 2$ average pooling. Global average pooling is applied for classification, followed by a softmax classifier. This leads to a compact model architecture. DenseNets consistently improve accuracy as the number of input parameters increases, without any signs of performance degradation or overfitting. Furthermore, DenseNets achieve good accuracy with significantly fewer parameters and computational resources. This effectiveness stems from their ability to extract essential features by leveraging condensed internal representations and reducing feature redundancy. Its architecture, as shown in Fig. 3, has been widely adopted, and serves as a basis for many other deep learning models.

#### Channel attention

**Fig. 4:**
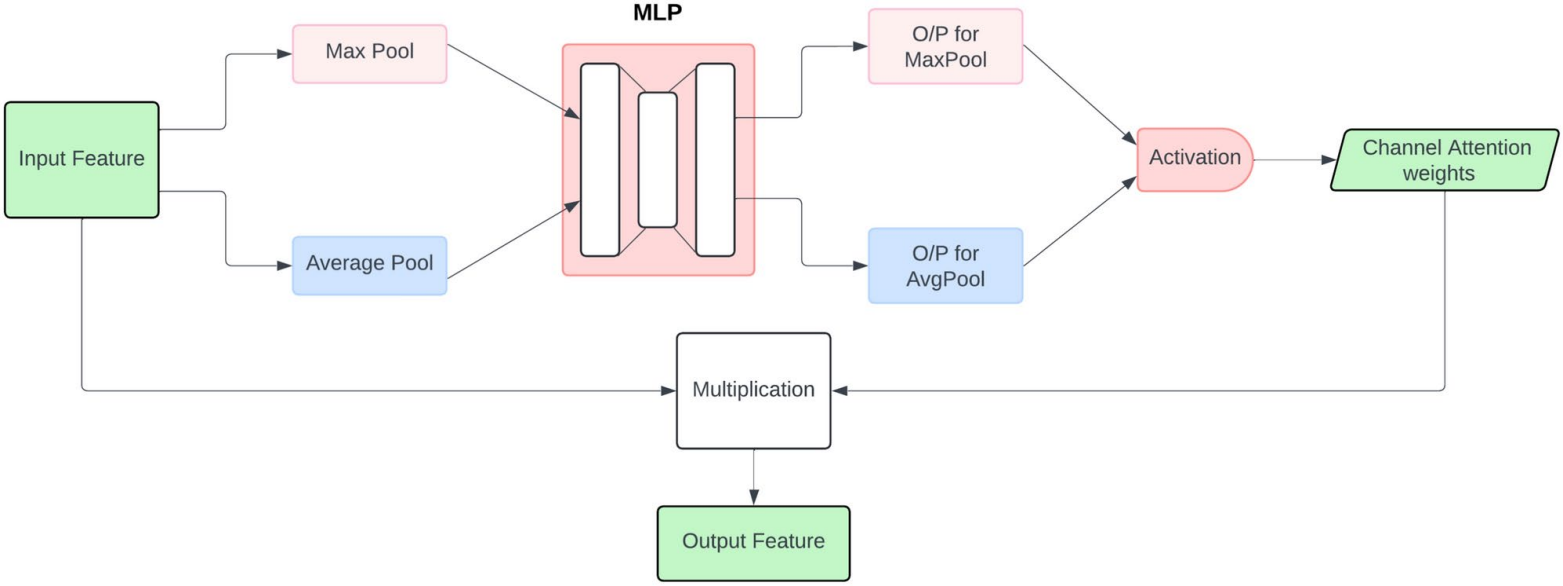
An illustration of the channel attention mechanism.

Attention plays a key role in human perception, especially within the visual system, where instead of attempting to process an entire scene at once, humans focus on specific, important areas to effectively capture visual details. This concept has been adopted in deep learning models, where attention mechanisms allow the network to concentrate on the most significant parts of the input, such as certain regions of an image. In the code provided, we incorporate this idea using a channel attention mechanism. Figure 4 illustrates the steps involved in this process.

The channel attention mechanism works by utilizing the inter-channel dependencies of the feature maps. Since each channel functions as a detector of specific features, applying channel attention enables the model to enhance the most relevant features in the input image. In the proposed solution, after extracting features using DenseNet121, the channel attention layer emphasizes critical channels, guiding the network to focus on meaningful information. To efficiently compute channel attention, the spatial dimensions of the input feature map are reduced through a global pooling operation, ensuring that the model processes only the most essential features from each channel.

We begin by extracting spatial information from the feature map through two pooling operations: average pooling and max pooling. These processes yield two separate spatial context descriptors, denoted as $F^{c}_{avg}$ for average-pooled features and $F^{c}_{max}$ for max-pooled features. Both descriptors are then passed through a shared network to generate a channel attention map $M_{c} \in \mathbb {R}^{C\times 1\times 1}$.

The shared network consists of a multi-layer perceptron (MLP) with one hidden layer. To minimize the parameter overhead, the hidden layer size is set to $\mathbb {R}^{C/r \times 1 \times 1}$, where *r* is the reduction ratio. Once the descriptors have passed through the MLP, the resulting output feature vectors are combined using element-wise summation. Finally, the channel attention is computed as:

$$\begin{aligned} A_{c}(F)&= \sigma (\text {MLP}(\text {GlobalAvgPool}(F)) + \text {MLP}(\text {GlobalMaxPool}(F))) \\&= \sigma (V_1(V_0(F_{avg}) + V_1(V_0(F_{max}))) \end{aligned}$$

In the expression, $A_{c}(F)$ represents the channel attention map generated from the input feature map $F$. The terms $F_{avg}$ and $F_{max}$ denote the feature maps obtained from global average pooling and global max pooling, respectively. $\text {MLP}$ refers to the multi-layer perceptron used for processing the pooled feature maps. $V_0$ and $V_1$ are the learnable weight matrices of the MLP. Finally, $\sigma$ represents the sigmoid activation function, while $\text {GlobalAvgPool}$ and $\text {GlobalMaxPool}$ indicate the global average pooling and max pooling operations.

For our work, we use DenseNet121^10^ model pre-trained on the ImageNet^43^ weights. The layers in DenseNet121 have been kept frozen and the trainable layers include the channel attention module, flattened layer, two dense layers and the output softmax layer. The attention layer is applied after the final convolutional layer of the pre-trained DenseNet121 model. The weights obtained after applying channel attention on the input feature is multiplied with it to obtain the output feature map. The output of the channel attention layer is then flattened, and fed into two fully connected dense layers following which we obtain the classification results.

We train the model for 25 epochs with a learning rate of 0.01. Adam optimizer is used along with the calculation of cross-entropy loss. After training the models for 25 epochs, the model weights are saved and later loaded in evaluation mode to generate feature space for validation and test sets. For feature generation we extract the pre-final layer dense layer.

### Feature selection

A vast dimension of features may be generated by the feature extraction process of any CNN model, which the classifier must process. However, all these features may not be useful for the classification tasks. The remaining elements are superfluous or irrelevant and simply serve to lengthen computation times and take up more physical space. Furthermore, the classification accuracy is lowered by the existence of these non-informative features. On the set of features generated from the aforementioned CNN model, feature selection has been conducted in order to eradicate the said issues. In the proposed method, the fitness of each chromosome in the GA population is evaluated using two distinct filter approaches, making the GA an adaptive feature selection algorithm.

#### Filter method

To calculate the fitness the individual chromosomes, we rely on the filter-based method, namely Minimum redundancy maximum relevance (mRMR).

*Minimum redundancy maximum relevance* mRMR^44^ is a filter ranking strategy, which ranks features according to correlation to the class and itself. Preferably, features having a low correlation among themselves and a strong correlation with the class (output) are picked. The F-statistic values^45^ for continuous features can be used to assess correlation with the class (relevance), while PCC values can be used to assess correlation between features (redundancy). The F-statistic is calculated by comparing the variance between classes (numerator) with the variance within each class (denominator). It evaluates how well a feature separates class labels, with a higher F-statistic indicating stronger relevance. Conceptually, this compares a “full model,” where the feature is included, to a “reduced model,” where it is excluded. The between-class variance represents the variability explained by the feature (full model), while the within-class variance reflects the residual variance (reduced model). Thus, the F-statistic measures the added value of including the feature in terms of class separation.

The features are chosen one at a time using a greedy search strategy in order to maximise the objective function, which is based on relevance and redundancy. The objective function that indicates the difference between relevance and redundancy, or the quotient of relevance and redundancy is computed via the formula -

$$score_{i}(f)= \frac{F(f,target)}{\sum _{s\in f'(i-1)} |corr(f,s)|/(i-1)}$$

Where i represents the i-th iteration, f is the feature under evaluation, F signifies the F-statistic^45^, f’(i-1) corresponds to the features chosen through (i-1) iterations, and “corr” indicates the Pearson’s correlation.

#### Overview of the genetic algorithm

**Fig. 5:**
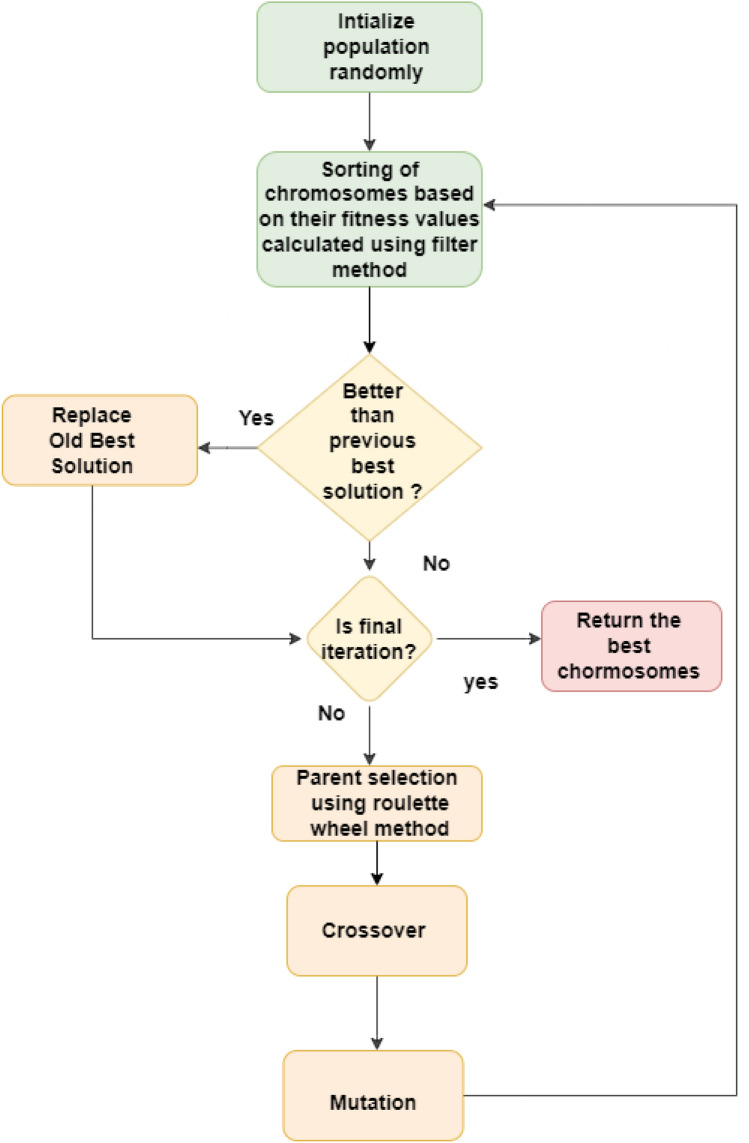
Various stages of the genetic algorithm.

GA^12^ is a well-known meta-heuristic evolutionary algorithm, which is utilized to tackle challenging optimization issues. It is a biologically based algorithm featuring selection, crossover, and mutation properties. Initial population development, parent selection, crossover, mutation, and generation of child chromosomes are all processes in GA. A random population is first created, each chromosome having a finite number of random values of a set length. From this collection of chromosomes, parent chromosomes are chosen, and after crossing and mutation, they are used to produce the offspring chromosomes. In the process of GA, the quality of the solution obtained in each iteration is evaluated by its fitness scores. This process is iteratively repeated, and new sets of chromosomes are generated using selection, crossover, and mutation operations. Individuals with lower fitness are eliminated to make room for new offspring in the subsequent generations.The flowchart consisting of various steps of the GA is shown in Fig. 5 . The application of genetic algorithm results in a solution that is close to optimal after a predetermined number of iterations. In FS, a binary version of GA is employed, where each chromosome is represented as a vector consisting of ’0’s and ’1’s. A ’0’ indicates the feature is not selected, while a ’1’ indicates the feature is selected.

#### Proposed GA variant

GA is an evolutionary algorithm inspired by nature, and it is one of the oldest and most widely used algorithms in the field of FS and optimization. Researchers have utilized GA to achieve near-optimal subsets of features from a given feature space. The algorithm employs key operators such as crossover and mutation to explore and exploit the search space. These operators enable GA to provide a balanced approach between exploration and exploitation. In order to achieve near-optimal solutions, numerous modifications have been suggested by researchers to improve GA. One challenge in GA is the randomness of the mutation probability. Additionally, the fitness of each candidate solution is determined by a learning algorithm, which can be computationally expensive. To address these issues, we propose an adaptive version of GA that estimates fitness by aggregating the results of three filter-based methods. This modification significantly reduces computational time. In addition to the fitness estimation modification, we propose a different fitness score value calculation method that improves the fitness of individual candidate solutions. We use a multi-point crossover and Roulette wheel parent selection to achieve better exploitation. Algorithm 1 provides the pseudo code for this fitness score value calculation technique.

**Algorithm 1:**
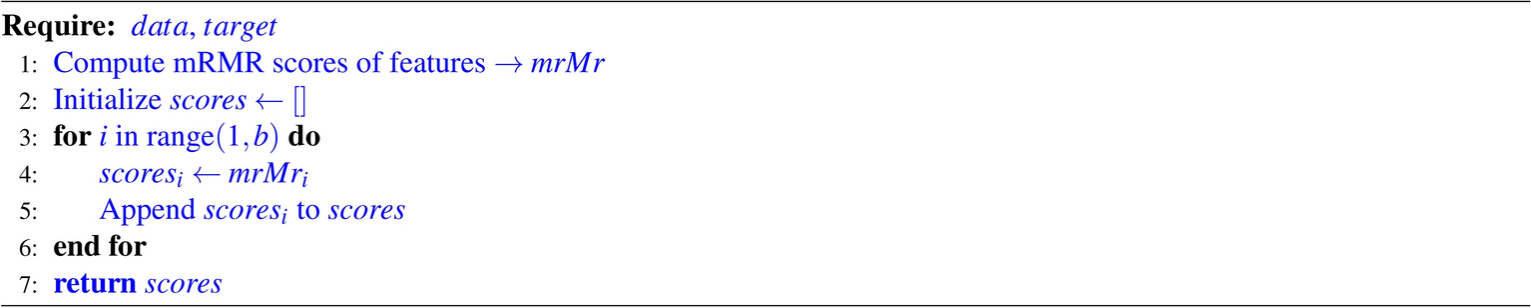
Score Calculation (using mRMR)

#### Fitness function

Wrapper-based FS methods typically employ a learning algorithm, such as a classifier, to evaluate the fitness of chromosomes. Although GAs are widely used as wrapper-based methods, they tend to increase computational time. To address this issue, in the present work, the use of classifiers is substituted with filter methods to determine the score of each feature vector (i.e., a chromosome). This approach helps assess the strength of each chromosome using a less expensive way, thereby reducing the overall computational complexity of the feature selection method. The algorithm for calculating the mRMR score for a chromosome, provided in Algorithm 1, is utilized by the fitness computation module (Algorithm 2) which is explained below . This module uses the mRMR scores to evaluate the relevance and redundancy of each selected feature.

Here, we provide the population size, maximum number of iterations, and random states (random seed for reproducibility) as parameters. Then from the feature set, we take an initial population on a random basis with varied dimensions. Next, we pass this initial population no of generations which is numerically equivalent to the max number of iterations. Inside the loop, we use a method that sorts the population on the basis of the fitness value of every chromosome which is previously determined for every chromosome by using the filter methods. This step ensures that solutions with better fitness values are given higher priority. A chromosome is basically a binary vector, where ’0’ denotes that the feature should not be included, and ’1’ signifies that the feature should be included for fitness calculation. Accuracy is used as part of the fitness value calculation to assess how well a specific subset of features contributes to the model’s predictive performance. Agents are evaluated using the fitness function, which considers their accuracy (and potentially other factors) to determine their quality. The population of agents evolves through selection, crossover, and mutation to optimize feature subsets over generations. By employing filter method, we obtain a value (i.e., a score) for each feature. It can be said that the feature column with the highest value is the most important, while the one with the lowest value is the least important. Consequently, to compute the score of each individual chromosome, we take the mean of these values for all features marked as ’1’. The pseudo code for the fitness value calculation is provided in Algorithm 2.

**Algorithm 2:**
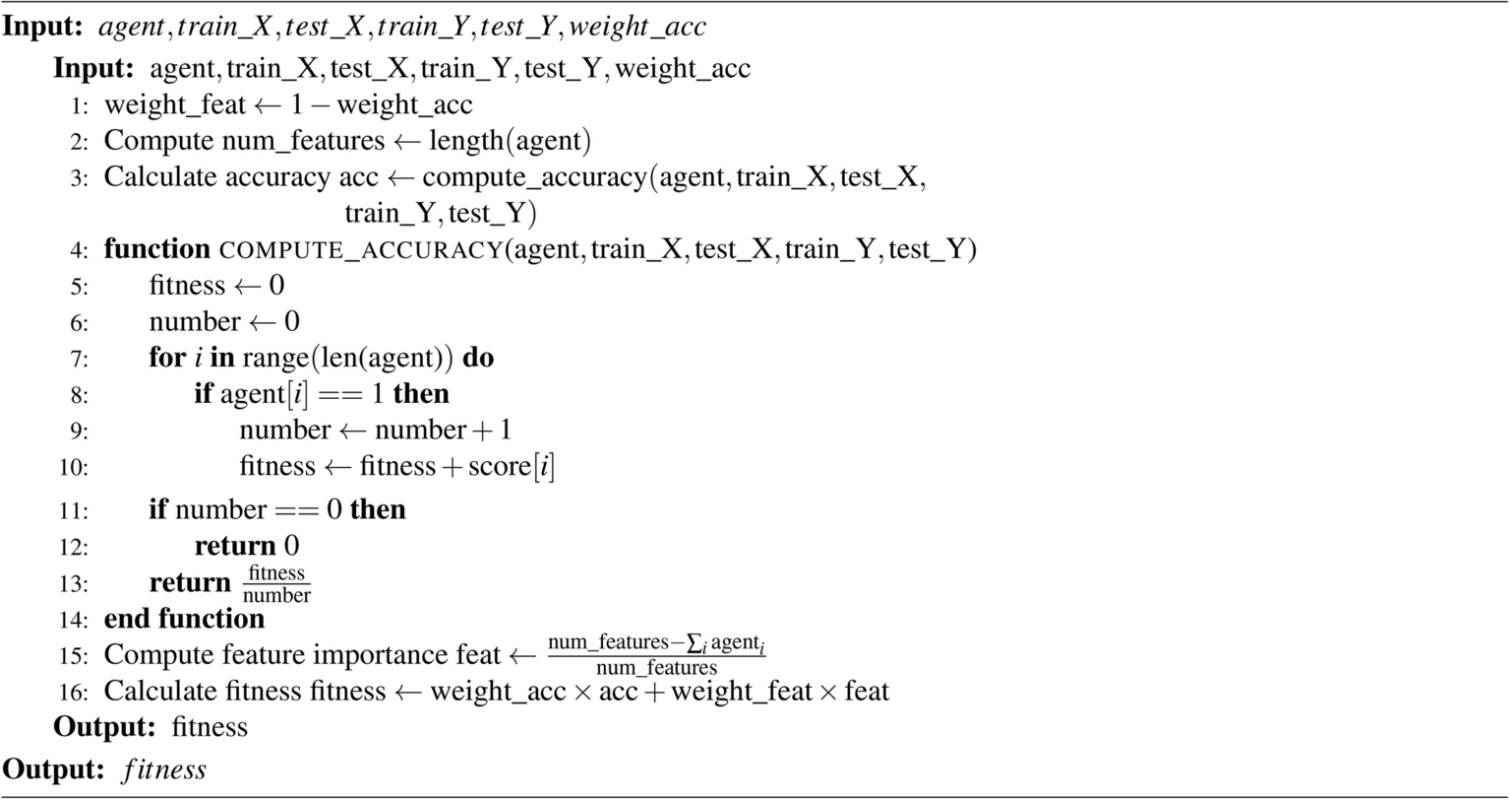
Compute Fitness

In FS, our goal is to simultaneously enhance the classification accuracy of the problem at hand and reduce the number of selected features. To achieve this, we introduce a single objective function that evaluates the overall fitness of each chromosome (feature subset). This objective function is defined in the following equation-

$$\text {fitness} = \alpha \cdot \text {acc} + (1 - \alpha ) \cdot \left(\frac{\text {num} \_\text {features} - \sum _{i} \text {agent}_i}{\text {num} \_\text {features}} \right)$$

where the term $\alpha$ represents the weight assigned to the accuracy component of the fitness score, while $(1 - \alpha )$ represents the weight assigned to the feature importance component. The variable $\text {acc}$ refers to the accuracy achieved by the agent, which is calculated using both the training and test sets. ${\text {num}}\_{\text{features}}$ denotes the total number of features being considered by the model, equivalent to the length of the agent vector. The sum $\sum _{i} \text {agent}_i$ corresponds to the number of features selected by the agent, where $\text {agent}$ is a binary vector indicating feature selection (1 for selected features, 0 for unselected features).

The following are the major stages of GA:

*1. Selection*: In GA, the selection process determines which individuals from the current population will become parents for the next generation, prioritizing those with higher fitness values for increased chances of selection. Here, the selection process is performed using Roulette Wheel Selection. The fitness values of the solutions are normalized, and selection probabilities are calculated based on the fitness values. A random number is then generated, and a solution is selected with a probability proportional to its fitness value. This process is repeated to select pairs of parents for crossover.

*2. Crossover*: In GA, crossover combines genetic information from two parent solutions, exchanging features to create new offspring and explore different regions of the solution space. Here, the crossover operation is performed between pairs of parent solutions (‘parent1’ and ‘parent2’). A crossover probability (‘crossoverprob’) determines the likelihood of performing the crossover operation. For each feature in the solutions, a random number is generated, and if it is less than the crossover probability, the corresponding feature values are swapped between the parents, creating two new child solutions (‘child1’ and ’child2’).

*3. Mutation*: In GA, mutation introduces random changes to individuals, adding diversity to the population and preventing premature convergence by exploring new areas of the solution space. Here, the mutation is applied to each solution in the population after the crossover operation. The mutation probability (‘mutationprob’) increases linearly with the current iteration. For each feature in the solution, a random number is generated, and if it is less than the mutation probability, the feature value is flipped (from 0 to 1 or from 1 to 0). The mutation process introduces random changes to the solutions, allowing the algorithm to explore new solutions that may have been missed through selection and crossover operations.

By combining selection, crossover, and mutation, the GA explores and exploits the population of solutions over multiple generations. Solutions with better fitness values have a higher chance of being selected, and genetic material is exchanged and mutated to explore different regions of the solution space. If better than the previous best solution is found in any solution then the old best solution is replaced by the new best solution. This iterative process continues until the maximum number of iterations is reached or a termination condition is met, ultimately converging toward an optimal or near-optimal solution. After iterations, the best solution (i.e., the best chromosome) is considered as the optimal feature subset, which is then fed to the KNN classifier.

### Classification

The feature selection step is followed by classification for which we have used a k-Nearest Neighbors (KNN)^46^ classifier.

#### KNN classifier

The KNN classifier is a non-parametric, instance-based learning algorithm that classifies a data point based on the majority class of its nearest neighbors in a feature space. Given a positive integer k and a query sample, the k-NN algorithm identifies the k training examples that are closest to the sample based on a distance metric (typically Euclidean distance). The query sample is then assigned the label that is most frequent among the k nearest neighbors. The k-NN classifier assumes that similar data points exist in close proximity in the feature space, making it suitable for pattern recognition and classification tasks where decision boundaries are complex and non-linear. This method requires no explicit training phase, and its performance is dependent on the value of k, the choice of distance metric, and the underlying distribution of data.

For our work we have set the k value to 5. The classifier was fitted using the train data generated by the GA applied on the extracted features, following which the fitted model was tested based on the test dataset.

## Experiments and results

### Performance metrics

In this paper, we use accuracy, precision, recall, and F1 score as the main performance metrics, along with the number of features and graphs, including confusion matrices, as performance measures.

*1. Accuracy*: The ratio of correctly predicted samples to the total. True Positive (TP): correctly predicted positive cases; True Negative (TN): correctly predicted negative cases; False Positive (FP): incorrectly predicted positive cases; False Negative (FN): incorrectly predicted negative cases. Accuracy is calculated as:

$$\text {Accuracy} = \frac{{TP + TN}}{{TP + TN + FP + FN}}$$

*2. Precision*: The percentage of correctly identified positive samples among all predicted positive samples:

$$\text {Precision} = \frac{{TP}}{{TP + FP}}$$

*3. Recall*: The proportion of actual positive samples correctly identified:

$$\text {Recall} = \frac{{TP}}{{TP + FN}}$$

*4. F1-score*: F1-score is a comprehensive approximation of the modelâ€™s performance, and it is nothing but the harmonic mean of precision and recall. It can be calculated using the following formula:

$$\text {F1-score} = \frac{{2*Precision*Recall}}{{Precision+Recall}}$$

*5. Confusion matrix*:The confusion matrix is a square matrix used to evaluate the performance of a classification model. It provides a visual representation of the model’s predictions by comparing the true class labels with the predicted class labels. The matrix consists of rows and columns, where each row represents the true class label instances and each column represents the predicted class label instances. The diagonal elements of the matrix indicate the number of correct predictions, where the predicted label matches the true label. The confusion matrix is a valuable tool for assessing the classification performance of a model.

### Results

In order to identify the optimal feature extractor, we tested several CNN models based on transfer learning (pretrained on ImageNet) on the dataset. The models compared include VGG16, DenseNet121, and Inception V3. Among these, DenseNet121 yielded the highest accuracy. Table 2 presents a comparison of the accuracies of these models. Additionally, we evaluated VGG19, ResNet50, Xception, and Inception ResNet V2. While VGG19 demonstrated high training accuracy (98.23%), its test accuracy was lower (95.42%). ResNet50, Xception, and Inception ResNet V2 showed poorer performance, with ResNet50 having the lowest test accuracy (78.55%).

Table 3 illustrates the precision, recall, and F1-score comparisons of the proposed model.

Table 4 provides a 5-fold cross-validation accuracy comparison among the tested models. DenseNet121 achieved the highest mean accuracy (97.6%), surpassing VGG16 and Inception V3, both of which attained a mean accuracy of 96.2%. Standard deviations were 0.4% for VGG16 and Inception V3, and 0.3% for DenseNet121, indicating consistent performance. The additional models did not outperform DenseNet121. Table 5 presents a performance comparison of the standard DenseNet model, DenseNet with an additional MLP layer, and DenseNet enhanced with Channel Attention, highlighting their respective accuracies and feature counts. We have also tested performance of using feature selection with the KNN classifier and our proposed methodology. The results of the comparison are shown in Table 6.

**Table 2:** Performance comparison of different CNN models tested on lung histopathological images with 25 epochs and cross-categorical entropy as the loss function. Accuracy scores are in %.

Sl No.	Model	Train Acc	Test Acc
1	VGG16	97.04	96.01
2	**DenseNet121**	**97.99**	**97.20**
3	Inception V3	96.47	96.10
4	VGG19	98.23	95.42
5	ResNet50	83.74	78.55
6	Xception	90.05	83.36
7	Inception ResNet V2	94.13	92.87

**Table 3:** Performance comparison of proposed model on various classes of lung cancer images in terms of Precision, F1-score, and the Recall.

Class	Precision	F1-Score	Recall
Lung adenocarcinoma	99.75	99.59	99.45
Benign lung tissue	100.0	100.0	100.0
Squamous cell carcinoma	99.45	99.66	99.80
**Average**	**99.73**	**99.75**	**99.75**

**Table 4:** 5-fold Cross-validation Results for CNN Models.

Model	Fold accuracy (%)				
	1	2	3	4	5
VGG16	96.5	95.8	96.2	95.9	96.7
DenseNet121	97.2	97.6	97.9	97.4	97.8
Inception V3	96.1	96.3	95.8	96.6	96.1
Mean accuracy (%)					
VGG16	96.2 ± 0.4				
DenseNet121	97.6 ± 0.3				
Inception V3	96.2 ± 0.4

**Table 5:** Performance comparison of DenseNet, DenseNet with one MLP layer, and DenseNet with Channel Attention.

Method	Accuracy (%)
DenseNet	97.20
DenseNet with one MLP layer	97.31
DenseNet with channel attention	98.95

**Table 6:** Performance comparison between KNN classifier trained on original DenseNet features and the proposed GA-based KNN classifier.

Method	Accuracy (%)	Precision (%)	Recall (%)	F1 Score (%)
KNN on original DenseNet features	97.45	96.80	97.10	96.95
Proposed GA-based FS with KNN classifier	99.75	99.60	99.65	99.60

**Table 7:** Performance comparison of model with only DenseNet 121 with Channel Attention, without FS, and with FS.

Method	Accuracy (%)	No. of features
Without FS	98.95	128
With FS	99.75	22

### Parameter tuning of GA

**Fig. 6:**
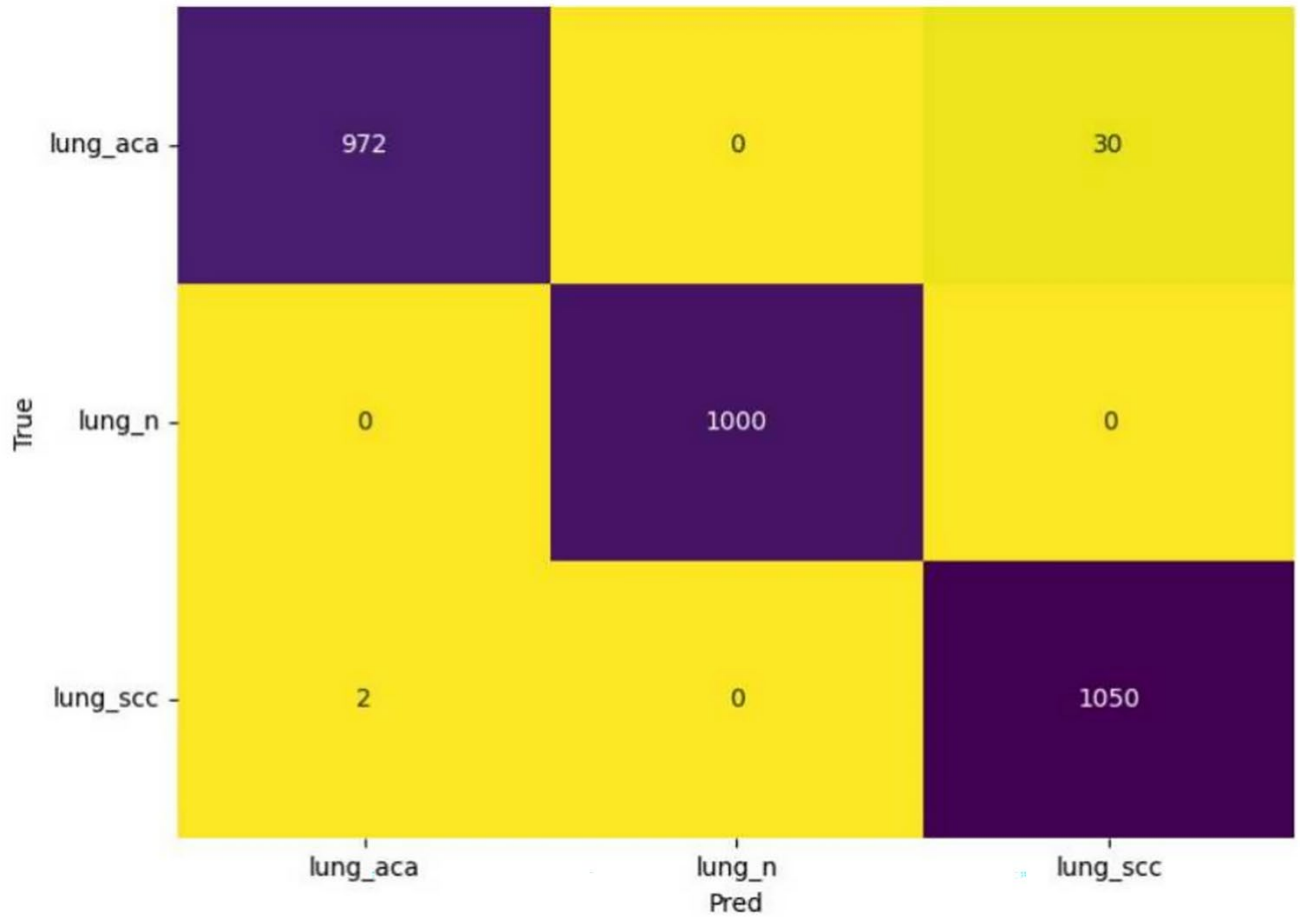
Confusion matrix obtained by DenseNet121 with channel attention on the LC25000 dataset i.e., without FS.

**Fig. 7:**
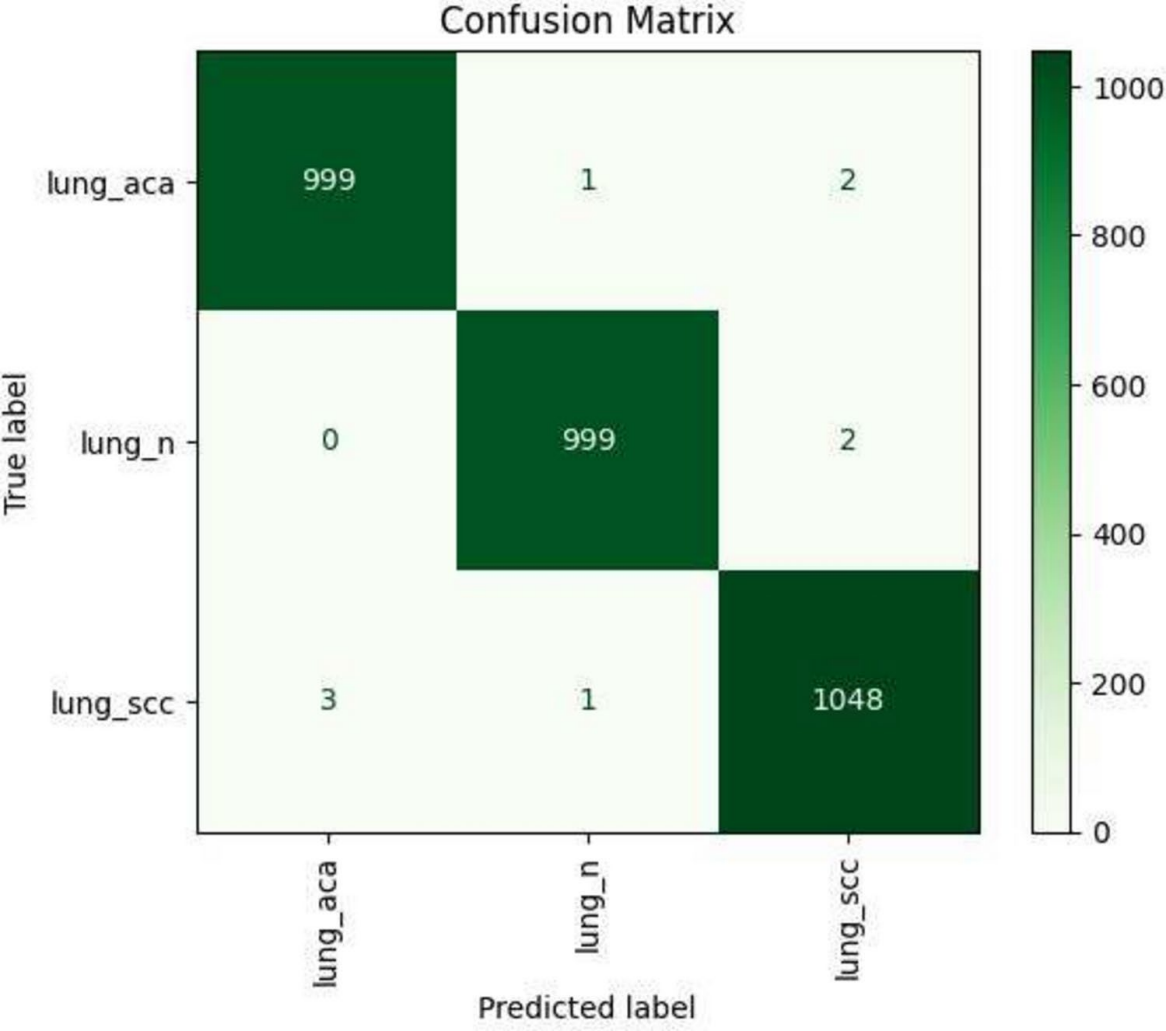
Confusion matrix obtained by the proposed method on the LC25000 dataset after applying FS.

To thoroughly ascertain the performance of the proposed model, we compare the accuracy of the model without FS and with FS. Upon comparison, we observe that the accuracy of the channel attention enabled DenseNet121 based model is 98.95%, while that with FS, it is 99.75% along with the fact that the number of features have reduced drastically from 128 to 22 as described in Table 7. This clearly shows that FS improves the accuracy of the overall model which can be supported further by comparing the Fig. 6 where have the confusion matrix obtained by the Channel attention enabled DenseNet121 model without FS with the Fig. 7 showing the confusion matrix for the model with FS..

The performance of the classification model is strongly influenced by the FS hyper-parameters. In this section, we analyze the impact of crucial FS hyper-parameters, namely population size, crossover probability, and the number of iterations, on the overall accuracy of the model.

#### Population size

**Fig. 8:**
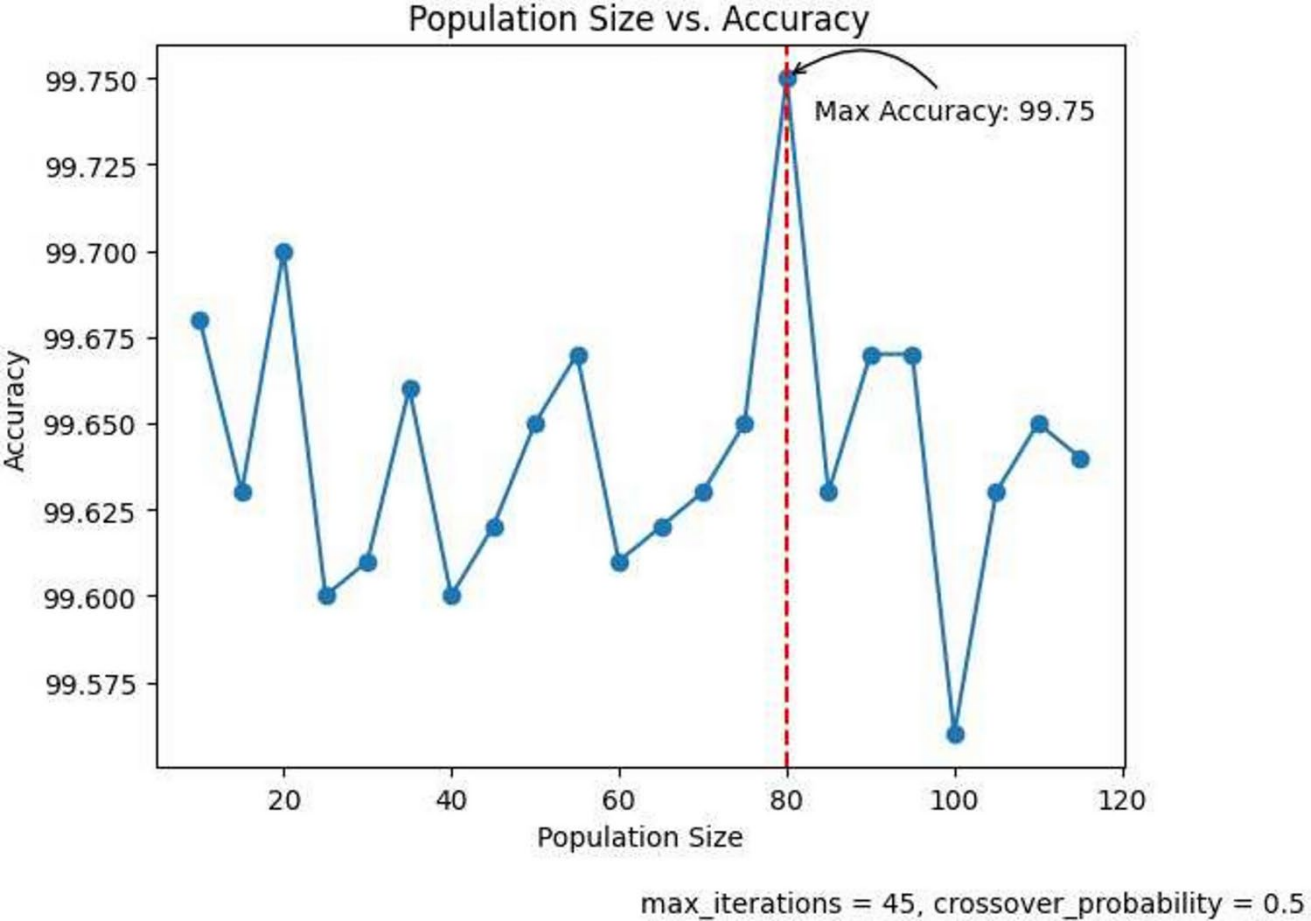
Population vs. Accuracy curve showing maximum accuracy of 99.75%.

The population size plays a crucial role in the search for the best solution within the given search space. A larger population size enhances the chances of finding an optimal solution. By increasing the population size, the exploration of the search space is expanded, allowing for a more comprehensive search and potentially leading to better results. In this paper, we experiment with different population sizes starting from 10 and increasing to 115 with a fixed interval of 5. The population vs accuracy graph is shown in Fig. 8. It is observed that the accuracy follows a zig-zag pattern with that maximum accuracy being 99.71% at a population size of 20. Hence we take 20 as the default population size for our proposed method.

#### Number of iterations

**Fig. 9:**
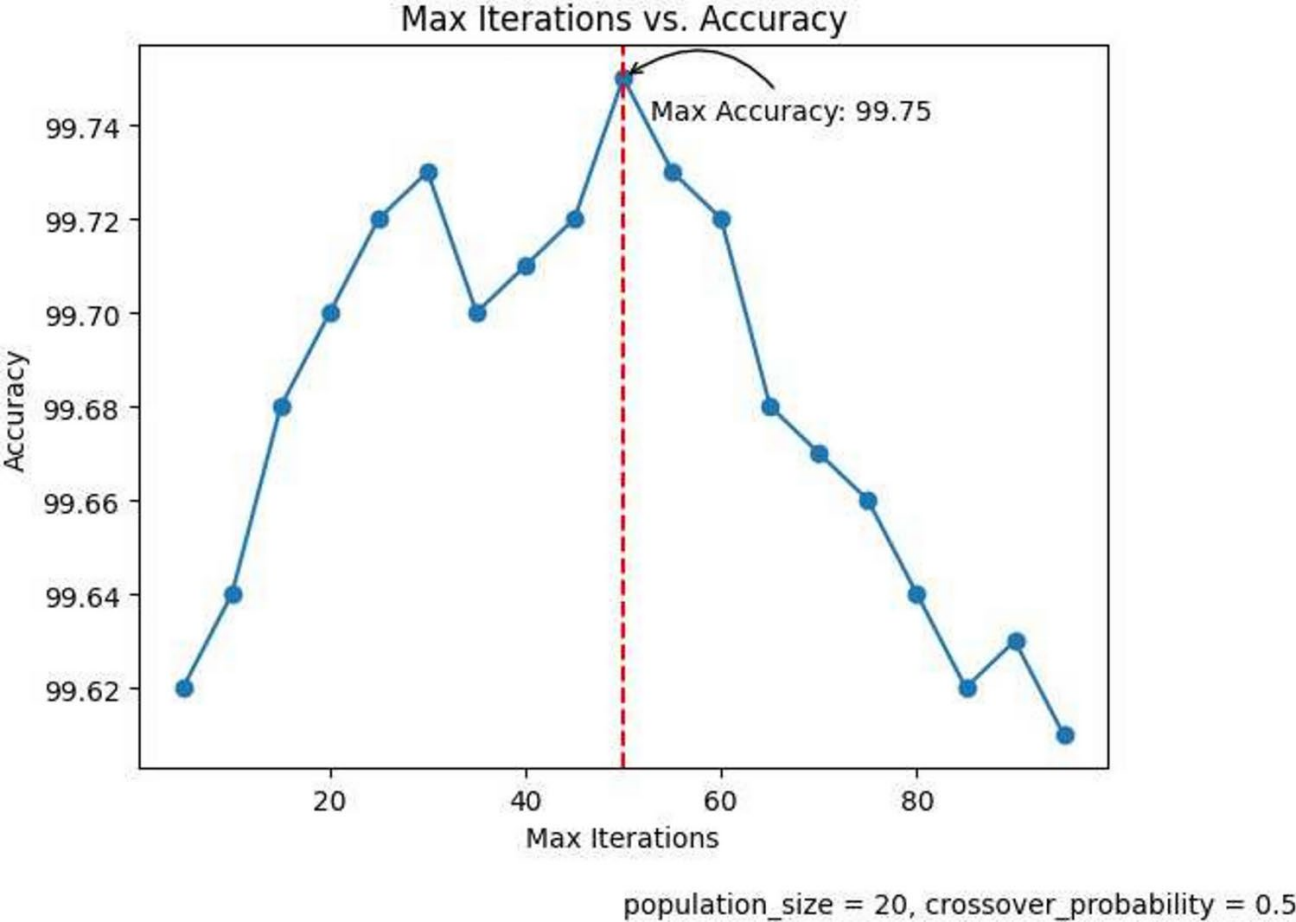
Number of iterations vs. Accuracy showing maximum accuracy of 99.75%.

**Fig. 10:**
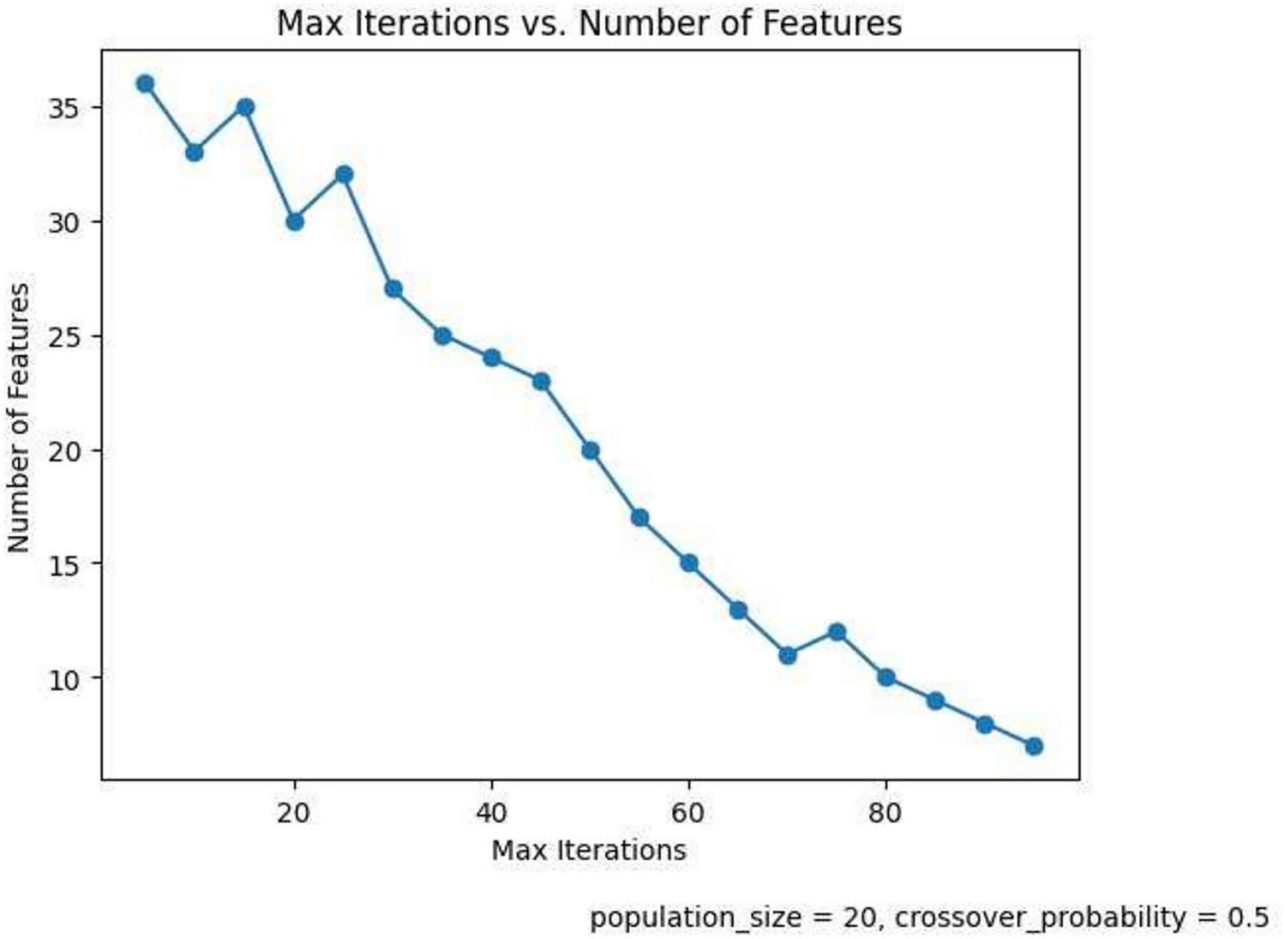
Number of iterations vs. Number of features in GA.

Figure 9 depicts the variation of the accuracy with the number of iterations. We start from 5 iterations and go up to 95 iterations. From the graph we observe that the accuracy ranges from 99.68 to 99.75 till 60 iterations and then registers a sharp fall. The maximum accuracy obtained is 99.75% at 50 iterations. Hence we take 50 iterations as the default number of iterations for our proposed method. Figure 10 shows the variation of the number of iterations with the number of selected features. The details of the hyperparameters selected at various stages of proposed method has been highlighted in Table 8.

#### Crossover probability

Crossover serves as a genetic operator that randomly generates fresh potential solutions by combining existing individuals within a population. The probability of crossover determines the chance of a mating event leading to a crossover. In this case, we have varied the crossover probability starting from 0.1 up to 0.95.

#### Minimum mutation probability

We perform mutation on the newly created offspring generated from crossover. For each offspring, the mutation is applied to randomly selected features. The probability of mutation is determined by mutation-prob, which gradually increases from min mutation probability to max mutation probability over the generations. In this case, we have varied the min mutation probability starting from 0.00 up to 0.10. The maximum accuracy achieved is 99.71% at a min mutation probability of 0.01. Hence we take 0.01 as the default min mutation probability for our work.

**Table 8:** Hyperparameter details of the various stages of our proposed method.

Stage	Hyperparameter	Value
Feature Extraction	Optimizer	Adam
	Learning rate	0.01
	No. of epochs	25
	Batch size	32
	Loss function	Categorical_crossentropy
Feature Selection	Population size	20
	Crossover probability	0.8
	No. of iteration	60
	Minimum mutation probability	0.01
	Maximum mutation probability	0.04
	Alpha	0.99
Classification	K value for KNN	5

### Comparison with state-of-the-art methods

Table 9 shows the performances of the past methods that had been applied on the LC25000 dataset. From the table, it can be concluded that our model shows better accuracy than the current state-of-the-art model while maintaining a low resource overhead.

**Table 9:** Comparison of the proposed method with past methods.

Model	Year	Accuracy (in %)
Mehmood et al.^18^	2022	89.90
Masud et al.^19^	2021	96.33
Mangal et al.^20^	2020	97.89
Hatuwal et al.^21^	2020	97.20
Nishio et al.^22^	2021	99.43
**Proposed**	**2024**	**99.75**

### t-SNE plots

t-SNE (t-Distributed Stochastic Neighbor Embedding) is a popular dimensionality reduction technique that can be used to visualize high-dimensional data in a lower-dimensional space. At first, the algorithm transforms the high-dimensional Euclidean distances among the data points into conditional probability scores that indicate similarities. This is achieved using SNE (Stochastic Neighbor Embedding) on the data points.

**Fig. 11:**
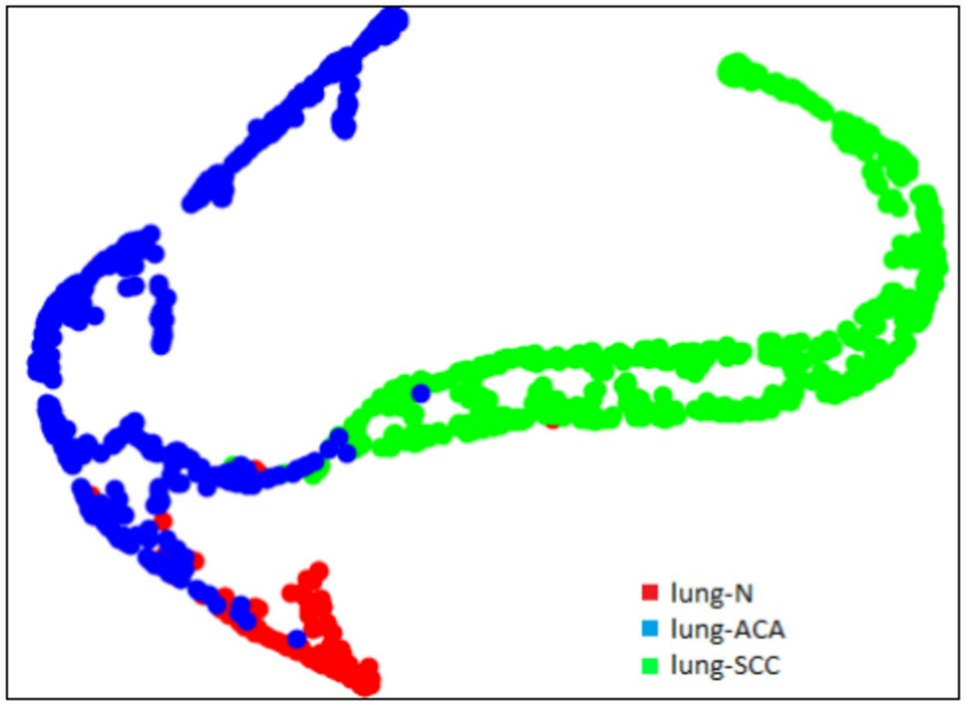
t-SNE plot of lung histopathological image samples obtained using the DenseNet121 model.

**Fig. 12:**
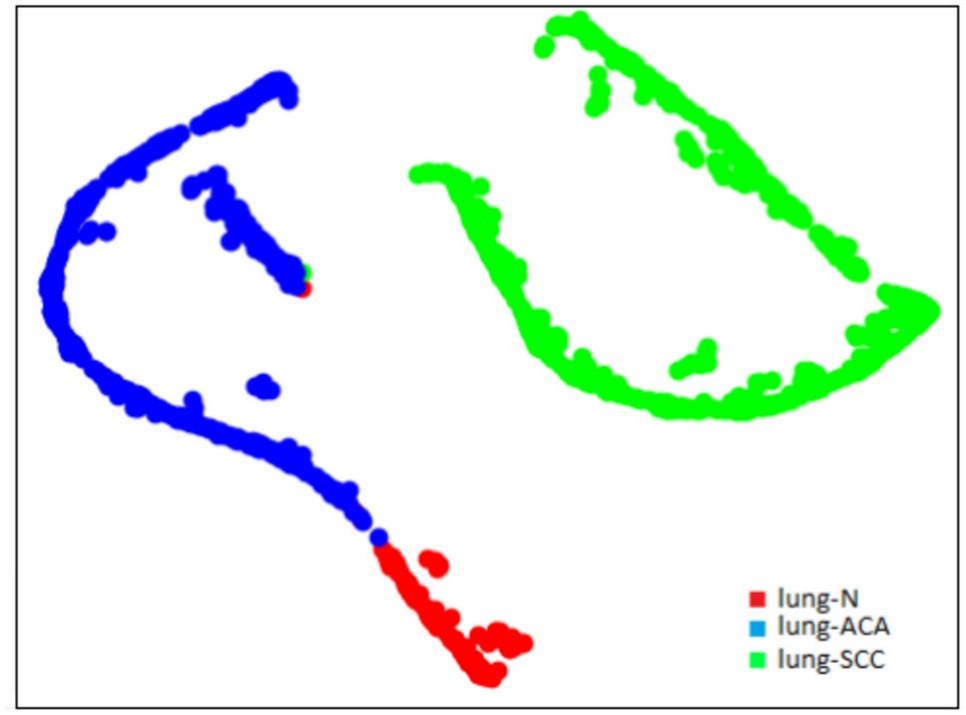
t-SNE plot of lung histopathological image samples using a combination of the DenseNet121 and GA.

We observe that the images corresponding to the three different classes of image from lung histopathological dataset are clearly separated into different clusters of samples. The t-SNE plot visualizations for the lung-n, lung-aca, and lung-scc classes, before feature reduction, are presented in Fig. 11. These visualizations have been generated using the DenseNet121 model. The t-SNE plot of the DenseNet121 model after feature reduction with GA is presented in Fig. 12.

### Statistical test

To test the hypothesis that the new algorithm produces outcomes similar to those of established algorithms, we have utilized the Wilcoxon Rank-Sum test^47^. We have selected some well-known FS algorithms and performed 15 separate executions for each algorithm on the employed dataset, documenting the classification accuracy of each run. Our set significance level is a p-value of less than 0.05. If the calculated p-value falls below this threshold, we have rejected the hypothesis due to insufficient evidence supporting the claim of comparability. Table 10 lists the p-values obtained for different FS algorithms. This statistically significant result suggests that the proposed algorithm consistently performs better than existing algorithms across different splits of the data, highlighting its stability and superior performance. Thus, we conclude that compared to the existing FS algorithms, our method demonstrates statistical superiority.

**Table 10:** p-values for different FS algorithms with our proposed method on LC25000 lung cancer images.

Algorithm	*p*-value
Grey wolf optimisation (GWO)	0.000197
Mayfly optimization algorithm (MA)	0.008389
Binary bat algorithm (BBA)	0.000750
Particle swarm optimization (PSO)	0.000068
Equilibrium optimizer (EO)	0.001199
Red deer algorithm (RDA)	0.000069
Whale optimization algorithm (WOA)	0.001592

### Results on the LIDC-IDRI dataset

In order to test the efficacy of the proposed method, we have also tested our method on another dataset, namely the LIDC-IDRI^48^ dataset. The dataset description can be found in Table 11, which shows the two classes - benign and malignant and also shows the number of images in each class. The accuracy achieved by the model before feature selection and after feature selection has been provided in Table 12. It clearly shows the improvement achieved by feature selection after feature extraction by DenseNet121 model, as opposed to only using DenseNet121. Further the efficacy and robustness of the proposed method can be verified by comparing its performance against state-of-the-art models based on the LIDC-IDRI dataset. From Table 13, it can be observed that the proposed solution performs comparably with the state-of-the-art models. Hence from these evidences, the robustness and efficacy of the proposed solution can be ascertained. Additionally, we have also tested the current proposed method using small cell lung cancer (SCLC) dataset, results of which are shown in Table 14. This table presents the results with SCLC histopathological images as training & validation dataset.

**Table 11:** LIDC-IDRI dataset description.

Image type	Benign	Malignant	Total
Train	4342	845	5187
Test	1340	282	1622
Validation	1073	224	1297
Total	6755	1351	8106

**Table 12:** Performance comparison of proposed method on LIDC-IDRI without FS and with FS.

Method	Accuracy (%)
Without FS	80.77
With FS	95.38

**Table 13:** Performance comparison of the proposed model with some existing methods for the LIDC-IDRI dataset.

Work Ref.	Method	Accuracy(%)
Rey et al.^49^	Fuzzy clustering, SVM and ANN	94.00
Jiang et al.^50^	Convolutional block attention module	91.00
Shaffie et al.^51^	Resolved ambiguity local binary pattern	92.50
Xie et al.^52^	Semi-supervised adversarial classification mode	92.50
Zhang et al.^53^	Basic 3D CNN, multi-output network, 3D DenseNet	90.40
**Proposed**	**Deep FS algorithm**	**95.38**

**Table 14:** Performance comparison of proposed model on various lung cancer datasets.

Dataset	Accuracy (%)	Precision (%)	Recall (%)	F1-Score (%)
NSCLC	99.75	97.66	99.12	98.38
SCLC	91.54	90.56	92.10	91.34
Combined (NSCLC + SCLC)	97.89	96.45	97.66	98.56

## Discussion

To make a fair idea about any new model, it’s required to know both its strengths and weaknesses. In this section, we discuss the key advantages of the proposed method as well as some cases where our method needs further attention.

### Underlying mechanisms

The strength of this method lies in the synergy between deep feature extraction and GA-based feature selection. DenseNet121, a CNN with dense connectivity, effectively captures high-dimensional patterns in medical images. The integration of the channel attention mechanism allows the model to emphasize essential image features, reducing noise and enhancing focus on relevant information for classification. This attention mechanism mimics human visual focus and contributes to the substantial increase in accuracy.

The GA further boosts model performance by optimizing feature selection. Using filter methods like mRMR, the GA evaluates feature effectiveness without the need for an expensive classifier. As a result, the number of features is reduced significantly, from 128 to 22, without sacrificing accuracy. The lightweight and efficient KNN classifier complements this approach by enabling fast and accurate classification using a smaller feature set.

### Comparative strengths of the proposed method

This approach offers significant advantages over previous methods. Achieving an accuracy of 99.75%, it outperforms the results of Mehmood et al. (89.9%) and Mangal et al. (97.89%). The success is largely due to the combination of the DenseNet121 model with channel attention, which enhances feature extraction by focusing on the most relevant parts of the image. Earlier methods lacked these advanced feature selection techniques.

Furthermore, the use of an adaptive GA dramatically reduces the feature space while maintaining accuracy. Unlike conventional wrapper-based GAs, which rely on classifiers for feature evaluation, this method uses filter-based techniques, making it more computationally efficient. The reduction of features from 128 to 22 illustrates the efficiency of our approach. In addition, the use of KNN further improves the model’s practicality by allowing rapid, scalable classification, making it well-suited for real-world applications with limited computational resources.

The application of GA in areas like satellite image classification and land cover classification is well-established, but its use in histopathological image analysis is relatively novel. This adaptation demonstrates the potential of GA as a powerful tool for reducing high-dimensional data in medical diagnostics, where redundant features can obscure important information.

### Challenges and limitations

Despite its benefits, the proposed method has some limitations. A key issue is the computational expense associated with Genetic Algorithms, particularly when applied to large datasets. As noted by Rao et al.^54^, GA performance can degrade as dataset size increases, leading to scalability issues. Although the adaptive GA in this study is more efficient than traditional wrapper-based GAs, it still requires significant computational resources, especially during feature ranking with mRMR.

Another challenge is the risk of local optima. Like many optimization techniques, Genetic Algorithms may become trapped in local optima, which can prevent them from finding the global optimal solution. This could negatively affect feature selection and classification performance. Additionally, random initialization of chromosomes and the use of genetic operators (such as crossover and mutation) can result in the generation of non-permutation matrices, which are invalid solutions. This increases the complexity of the optimization process, requiring more time and resources to address these challenges.

## Conclusion and future scope

In this paper, we have proposed a lung cancer classification model, where we combine the concepts of deep learning and FS algorithm. In our approach, an adaptive GA is applied to optimize the feature vector generated by a CNN model, and the proposed approach has been tested on the LC25000 dataset for detection of lung cancer. We have extracted the deep features from the histopathological images using channel attention aided CNN model for which we have selected the DenseNet121 model after testing multiple CNN architectures. The extracted features obtained have been passed through the adaptive GA based FS method, which efficiently reduces the feature space to $3/20^{th}$ of the original feature space. The optimal hyperparameters have been obtained after trials over a large range of values. Though the obtained results are satisfactory, however, there is no room for error in medical image analysis field. Hence, we have some future plans for making the model error-free. The scope for future work include proposal of a better feature extractor using deep learning models. Other filter methods may also be tried out in the adaptive GA approach that take less time to compute.

## Data Availability

A publicly available dataset for histopathology images was analyzed in this research work. The data can be found at: https://www.kaggle.com/datasets/andrewmvd/lung-and-colon-cancer-histopathological-images^55^ (accessed on 25 May 2023).
